# Supplementary material for: The L1014F Knockdown Resistance Mutation Is Not a Strong Correlate of Phenotypic Resistance to Pyrethroids in Florida Populations of Culex quinquefasciatus
Source: Insects. 2024 Mar 15;15(3):197. doi: 10.3390/insects15030197 (PMC10971530; doi:10.3390/insects15030197)
Supplement: Supplementary file 1 [file insects-15-00197-s001.zip › files submitted to AgDataCommons/Table S1.zip.pdf]

Table S1. Sample specific information for collections and data used in this study

Latitude	Longitude	County	Location	Collection Date	Provider/ Data source[1]
--	--	Laboratory	CMAVE Cxq	Jul-20	Eva A. Buckner
29.635	-82.360	Alachua	CMAVE	9/9/2020	Alden Estep
30.248	-85.895	Bay	Conservation	8/12/2020	Mike Riles
30.233	-85.878	Bay	Beach Parkway	8/12/2020	Mike Riles
25.992	-80.246	Broward	Shalimar	11/4/2020	Tamara Hamilton
25.986	-80.246	Broward	Tarpon	9/30/2020	Tamara Hamilton
26.014	-80.291	Broward	Pembroke Pines	10/8/2020	Tamara Hamilton
26.035	-80.177	Broward	Farragut	11/4/20, 3/24/21, 4/6/21	Tamara Hamilton
26.035	-80.178	Broward	34th Ave	11/4/20, 3/24/21	Tamara Hamilton
26.069	-80.208	Broward	SR-7	3/24/2021	Tamara Hamilton
30.013	-81.853	Clay	Middleburg	6/23/2020	Alden Estep
26.138	-81.796	Collier	Cambier Park	Jul-17	**Lucas et al 2020**
26.116	-81.759	Collier	Sugden Park	Jun-18	**Lucas et al 2020**
26.090	-81.726	Collier	Naples Manor	Jun-18	**Lucas et al 2020**
26.158	-81.658	Collier	Landfill	Aug-17	**Lucas et al 2020**
26.227	-81.672	Collier	Big Cypress Elem.	Jul-17	**Lucas et al 2020**
26.210	-81.546	Collier	Palmetto Elem.	Jul-17	**Lucas et al 2020**
26.265	-81.526	Collier	Sabal Palm Elem.	Jun-18	**Lucas et al 2020**
30.531	-87.307	Escambia	W 9 Mile Rd	4/11/2021	Kaylyn Cullen
30.539	-87.283	Escambia	Lyman	3/29/2021	Kaylyn Cullen
30.546	-87.308	Escambia	Amanda	4/8/2021	Kaylyn Cullen
28.493	-82.555	Hernando	Spring Hill	1/9/2020	S. Fisher-Grainger
28.031	-82.125	Hillsborough	Oak Lawn Cemetery	4/7/2021	Eva A. Buckner
27.609	-80.497	Indian River	Farms WCD	9/27/2020	Eva A. Buckner
28.805	-81.534	Lake	Mt. Plymouth	2021	Justin Walker
26.673	-81.932	Lee	Cape Coral	4/27/2020	Kara Tyler Julian
26.714	-81.843	Lee	Bayshore	7/8/2020	Kara Tyler Julian
26.642	-81.862	Lee	Ft Myers	7/28/2020	Kara Tyler Julian
26.641	-81.998	Lee	Bonefish Grill	3/22/2021	Kara Tyler Julian
26.675	-81.795	Lee	Pine Ridge Condos	3/19/2021	Kara Tyler Julian
26.614	-81.608	Lee	LA Golf Course	10/30/2020	Kara Tyler Julian
26.457	-82.050	Lee	Causey Ct	1/15/2021	Kara Tyler Julian
26.567	-81.849	Lee	Corporate Park	2/10/2021	Kara Tyler Julian
26.605	-81.959	Lee	Academy & Veteran	2/22/2021	Kara Tyler Julian
26.352	-81.796	Lee	Highland Woods	3/24/2021	Kara Tyler Julian
26.416	-81.795	Lee	Shadow Wood	4/7/2021	Kara Tyler Julian
26.370	-81.801	Lee	Bernwood Drive	4/9/2021	Kara Tyler Julian
26.682	-81.805	Lee	Big Lots	4/14/2021	Kara Tyler Julian

25.517	-80.493	Miami-Dade	SW 268th St	9/29/2021	**Unlu et al 2024**
25.896	-80.126	Miami-Dade	Park Dr	10/15/2020	**Unlu et al 2024**
25.930	-80.216	Miami-Dade	NW 170th Terrace	9/29/2020	**Unlu et al 2024**
25.862	-80.313	Miami-Dade	W 44th St	9/10/2020	**Unlu et al 2024**
25.814	-80.226	Miami-Dade	NW 42nd St	9/11/2020	**Unlu et al 2024**
25.834	-80.129	Miami-Dade	Alton Rd	10/14/2020	**Unlu et al 2024**
25.446	-80.456	Miami-Dade	SE 24th Ct	9/29/20, 10/1/20	**Unlu et al 2024**
25.968	-80.214	Miami-Dade	NW 7th Ave	9/29/2020	**Unlu et al 2024**
25.636	-80.372	Miami-Dade	SW 110th Ave	2020	**Unlu et al 2024**
25.637	-80.294	Miami-Dade	SW 144th St	8/21/20-9/2/20	**Unlu et al 2024**
25.790	-80.206	Miami-Dade	NW 6th Pl	7/30/2020	**Unlu et al 2024**
25.805	-80.196	Miami-Dade	NW 30th St	8/18/2020	**Unlu et al 2024**
25.853	-80.131	Miami-Dade	Bay Dr	8/12/20, 8/13/20	**Unlu et al 2024**
25.588	-80.313	Miami-Dade	SW 193rd Lane	8/31/20-9/02/20	**Unlu et al 2024**
25.754	-80.337	Miami-Dade	SW 87th Pl	9/8/20-9/9/20	**Unlu et al 2024**
25.657	-80.285	Miami-Dade	Moss Ranch Rd	8/5/2020	**Unlu et al 2024**
25.812	-80.416	Miami-Dade	NW 41st St	8/5/2021	**Unlu et al 2024**
26.703	-80.060	Palm Beach	North St.	9/9/2020	Steve Fazekas
26.932	-80.118	Palm Beach	Delaware	2/11/2020	Steve Fazekas
26.882	-80.144	Palm Beach	Donald Ross Rd	2/9/2021	Steve Fazekas
26.526	-80.184	Palm Beach	Boyton	3/16/2021	Steve Fazekas
26.530	-80.104	Palm Beach	Old Boyton	4/7/2021	Steve Fazekas
26.809	-80.200	Palm Beach	Ibis Reserve	3/16/2021	Steve Fazekas
26.655	-80.182	Palm Beach	Lyons	3/23/2021	Steve Fazekas
26.529	-80.123	Palm Beach	Military Trail	3/23/2021	Steve Fazekas
26.660	-80.265	Palm Beach	New Castle	2/23/2021	Steve Fazekas
26.654	-80.221	Palm Beach	Shower Tree	3/3/2021	Steve Fazekas
26.608	-80.107	Palm Beach	Villas Santorini	2/17/21, 3/30/21	Steve Fazekas
28.251	-82.741	Pasco	Gulfside	4/8/2021	Eva A. Buckner
28.223	-82.722	Pasco	Creek Pub	4/8/2021	Eva A. Buckner
28.245	-82.736	Pasco	Seaforest	2020	Agne Prasauskas
28.214	-82.719	Pasco	Mockingbird	2020	Agne Prasauskas
28.273	-82.690	Pasco	Orchid Lake	2020	Agne Prasauskas
28.273	-82.677	Pasco	Blueberry	2020	Agne Prasauskas
28.031	-82.656	Pinellas	Oldsmar Sewer	7/6/2020	Jason Stucke
28.113	-82.767	Pinellas	North Hwy	7/6/2020	Jason Stucke
27.847	-82.672	Pinellas	Sawgrass Lake	7/6/2020	Jason Stucke
27.975	-82.765	PInellas	Clearwater Nursery	7/6/2020	Jason Stucke
27.726	-82.651	Pinellas	Lake Maggiore	7/6/2020	Jason Stucke
27.829	-82.748	PInellas	Cross Bayou	7/6/2020	Jason Stucke
28.152	-82.663	Pinellas	Keller WTF	8/13/2020	Jason Stucke
27.879	-82.804	PInellas	Walsingham Park	8/13/2020	Jason Stucke
28.208	-81.575	Polk	Davenport	2020	Carl Boohene

28.061	-81.503	Polk	Kissimmee	2020	Carl Boohene
27.895	-81.473	Polk	Lake Wales	2020	Carl Boohene
28.004	-81.960	Polk	"E"	9/16/2020	Carl Boohene
27.896	-81.850	Polk	Tire Center	9/16/2020	Jackson Mosley
29.657	-81.446	St. Johns	Light	9/9/2020	Steven Peper
29.763	-81.312	St. Johns	6520 US Hwy 1	9/9/2020	Steven Peper
27.505	-80.358	St. Lucie	Fairwinds	6/15/2020	Sandy Jackson
28.871	-80.854	Volusia	S. County St.	9/9/2020	Miranda Tressler
30.385	-86.351	Walton	San Destin	8/13/2020	Cami Adams
30.321	-86.141	Walton	Odessa	9/9/2020	Cami Adams
30.320	-86.135	Walton	Savannah St.	9/9/2020	Cami Adams
30.337	-86.165	Walton	MONETMONET2	9/9/2020	Cami Adams
30.337	-86.165	Walton	MONETMONET	8/13/2020	Cami Adams
30.321	-86.139	Walton	Seaside	8/13/2020	Cami Adams
30.314	-86.088	Walton	Seagrove Beach	7/22/2020	Cami Adams

[1] Samples from previous Florida *Culex quinquefasciatus* resistance studies included in analyses in this manuscript (Lucas et al 2020, Unlu et al 2023)
